# Extratumoral Signs of Malignant Nonspiculate and Noncalcified Masses on Mammography: Are They Associated With Prognostic Factors in Breast Cancer?

**DOI:** 10.1155/tbj/2793342

**Published:** 2025-06-24

**Authors:** Ye Xu, Xinxin Yang, Fei Wang, Dongbo Wu, Jianghong Sun, Hongxue Meng, Xiushi Zhang

**Affiliations:** ^1^Department of Radiology, Harbin Medical University Cancer Hospital, 150 Haping Road, Harbin 150081, Heilongjiang, China; ^2^Precision Medical Center, Department of Pathology, Harbin Medical University Cancer Hospital, 150 Haping Road, Harbin 150081, Heilongjiang, China

**Keywords:** breast cancer, malignant breast nonspiculate and noncalcified masses, mammographic extratumoral structural abnormalities, mammography, nonspiculate and noncalcified masses, prognosis

## Abstract

**Aim:** To investigate the association between mammographic extratumoral signs, specifically their subclassifications, of nonspiculate and noncalcified masses (NSNCMs) and prognostic factors in breast cancer.

**Materials and Methods:** This retrospective study analyzed imaging and pathological data from 374 patients, categorizing extratumoral signs into structural abnormalities (parenchymal and trabecular) and halo, while also undergoing subclassification. The focus prognostic factors were achieved through screening. Then, univariate and multivariate analyses were performed. Correlation analysis was also employed to determine the relationship between subclassifications and prognostic factors.

**Results:** Lymphovascular invasion (LVI), Ki-67 levels, and stromal tumor-infiltrating lymphocytes (sTIL) levels were identified as the focus prognostic factors. Among tumor signs, only tumor margin was associated with sTIL levels. Extratumoral trabecular signs exhibited a significant correlation with LVI (OR = 2.5, *p*=0.007) and Ki-67 levels (OR = 1.23, *p*=0.001). Specifically, the parallel sign showed a positive correlation with LVI (*p*=0.009, *r* = 0.134), while the reticular sign displayed a positive correlation with Ki-67 levels (*p*=0.009, *r* = 0.134). Extratumoral parenchymal signs were found to be an independent predictor for sTIL levels (OR = 0.64, *p* < 0.001), with a negative correlation observed between the contraction sign and sTIL levels (*p* < 0.001, *r* = −0.185), as well as between the atrophy sign and sTIL levels (*p*=0.046, *r* = −0.103).

**Conclusion:** Specific extratumoral structural abnormalities of mammographic malignant NSNCMs showed a significant correlation with prognostic factors in breast cancer, warranting increased attention in research and clinical practice.

## 1. Introduction

The global incidence of breast cancer affects approximately 2 million individuals annually, making it a leading cause of cancer-related mortality among women worldwide [1–3]. The mammography has become one of the most significant diagnostic approaches for breast tumors in previous years [4, 5]. Considering the complex nature and high costs associated with MRI, mammography emerges as a more practical and cost-effective modality for evaluating breast masses, while also demonstrating superior spatial resolution. Population-based mammographic screening has been implemented in many countries and has shown a reduction in breast cancer mortality by up to 30% [6]. However, despite advancements in screening and treatment strategies, breast cancer remains a significant contributor to cancer-related deaths due to its high intratumor heterogeneity, which leads to variability in disease progression and treatment response. To enhance personalized prognostication and stratified decision-making, various biomarkers including tumor-infiltrating lymphocytes (TILs) have been proposed. Mammography not only enables the detection of breast cancer but also exhibits correlations with histopathological and immunological markers across diverse imaging findings [7–9]. Given the genomic and phenotypic heterogeneity of breast cancers, understanding these characteristics is crucial for prognosis determination and guiding therapeutic approaches [10, 11].

A breast mass is a common presenting feature of breast carcinoma [12] Among them, the nonspiculate and noncalcified masses (NSNCM) exhibit benign appearance but poor prognosis in some breast cancer cases, thus warranting further study. These studies primarily focus on signs of the mass itself, such as tumor shape or margin, which provide valuable decision-making aids for clinical practice [13–15]. However, there is limited literature available regarding the association between extratumoral structural abnormalities and prognostic factors in NSNCM using mammography imaging. Although mammography lacks quantitative analysis advantages, it effectively demonstrates the overall distribution and morphology of extratumoral structural abnormalities. Morphological analysis remains fundamental in imaging for both benign/malignant prediction and prognosis.

In this study, besides analyzing the characteristics of NSNCM themselves, our objective is to investigate the correlation between extratumoral structural abnormalities observed on mammography and prognostic factors in breast cancer based on our prior research findings regarding malignant mass prediction. This will provide valuable data support for prognosis prediction research based on mammography.

## 2. Materials and Methods

### 2.1. Ethic

This study was approved by the Institutional Review Board of Harbin Medical University Cancer Hospital (approval no. KY2022-67), and the requirement for informed consent was waived due to the retrospective nature of the research. To ensure patient confidentiality, all personally identifiable information—including names, hospital ID numbers, and imaging identifiers—was removed. Each case was assigned a unique anonymized code (e.g., “case001” and “case002”). All data were securely stored in isolated, access-controlled servers. Only authorized investigators had permission to access the data, and all access activities were logged and monitored in compliance with institutional data governance protocols.

### 2.2. Patients

A total of 2573 cases were initially retrieved using the keyword “mass” in the mammography report interface of the picture archiving and communication system (PACS) at our hospital. The search was restricted to inpatient cases between January and December 2018. The year 2018 was selected to ensure a consistent dataset with complete imaging, pathological, and surgical information, while avoiding inconsistencies and incomplete follow-up data present in more recent years.

Among the 2573 cases, 1025 (39.8%) patients were identified with postoperative pathology indicating “invasive ductal carcinoma.” Subsequently, 550 (21.4%) cases presenting spiculated or calcified masses were excluded due to their well-known association with typical malignant morphologies and distinct biological behavior [13–16].

**Figure 1:**
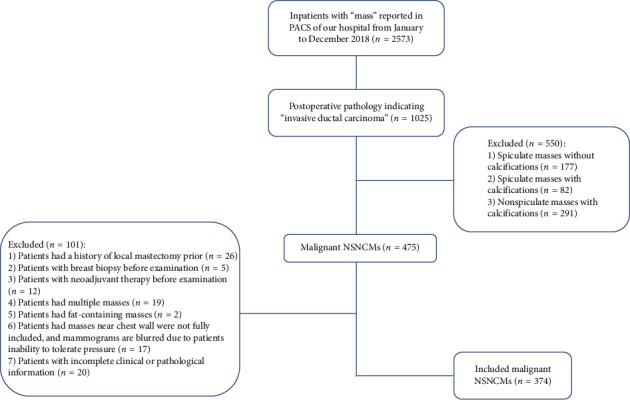
Flowchart of the enrolled study cases.

Additionally, 101 (3.9%) unsuitable cases were excluded from this study. Ultimately, a cohort of 374 female patients with surgically confirmed malignant NSNCM was included in our analysis (Figure 1).

### 2.3. Clinical and Pathological Data

Clinical and pathological data were systematically collected. Clinical parameters included age, body mass index (BMI), carcinoembryonic antigen (CEA), and cancer-associated antigen (CA) 153.

Pathological data were obtained from postoperative histopathological reports, and pathologists conducted a comprehensive review of the pathological images. Histopathological reports encompassed the histological tumor stage, tumor size, lymph node (LN) status, estrogen receptor (ER), progesterone receptor (PR), and human epidermal growth factor receptor 2 (HER2) expression, lymphovascular invasion (LVI), Ki-67 proliferative index (PI), P53 status, and stromal tumor-infiltrating lymphocytes (sTILs). These were assessed using the American Society of Clinical Oncology and the American College of Pathologist guidelines, employing immunohistochemical (IHC) methods [17–19].

According to recommendations from previous studies, the Ki-67 levels were classified as low (Ki-67 PI < 10%), intermediate (10%–30%), or high (> 30%) [20]. Similarly, the sTIL levels were categorized as low (sTILs 0%–10%), intermediate (11%–39%), or high (≥ 40%), following the International TILs Working Group guideline [21, 22].

Considering IHC results, tumors were classified as Luminal A-like (LA; ER/PR+ HER2−), Luminal B-like (LB; ER/PR+ HER2+), HER2−enriched (HER2; ER+ PR+ HER2+), and Triple negative (TN; ER− PR− HER2−), according to the 2013 St. Gallen International Breast Cancer Conference classification [23].

### 2.4. Digital Mammography Acquisition

Mammograms were obtained with the full field digital mammography system (Fuji MS-3500 and Siemens Inspiration). Experienced technologists performed the examinations in automatic exposure mode, with manual exposure mode utilized for larger masses. Conventional cranio-caudal (CC) and medio-lateral oblique (MLO) views were obtained for all patients, supplemented by additional positions when necessary. The examination pressure was adjusted based on effective communication to ensure maximum subject tolerance. All images were transmitted to both PACS and diagnosis workstation.

### 2.5. Imaging Analysis

Three radiologists independently reviewed all mammographic views on a specialized diagnostic workstation (5.8 M dual display screen) without prior knowledge of the pathological diagnosis. The imaging data were recorded by a radiologist with 7 years of experience in breast imaging. Subsequently, they were reviewed by a deputy chief physician who has been involved in mammography diagnosis for 18 years and, as well as a chief physician with an impressive track record of 20 years' involvement in mammography diagnosis. The three radiologists achieved consensus through thorough discussion to resolve any discrepancies in descriptors.

**Figure 2:**
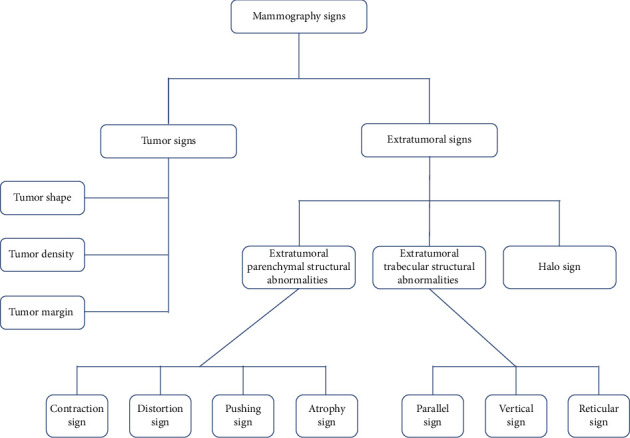
Classification map of mammographic tumor signs and extratumoral signs (including their subclassifications).

Tumor signs were assessed and documented according to the 2013 American College of Radiology (ACR) Breast Imaging Reporting and Data System (BI-RADS) lexicon [24]. Additionally, in a previous study conducted by our research team [25], extratumoral signs of malignant NSNCM were systematically classified according to BI-RADS guidelines and our experience in breast imaging. The classification methodology was provided in Figure 2 for further details.

### 2.6. Statistical Analysis

Firstly, to investigate which prognostic factors were most closely associated with mammographic findings, we conducted a univariate analysis between various prognostic factors and all mammography signs. A significance threshold of *p* < 0.01 was applied to select the key prognostic factors, which were subsequently identified as LVI, Ki-67, and sTIL. Next, for each of these prognostic factors, a second round of univariate analysis was conducted to examine their association with potential predictive variables (e.g., age, BMI, CEA, tumor size, and histological grade). Variables with *p* < 0.05 in this step were entered into multivariable logistic regression models to identify independent predictors.

All statistical analyses were conducted using R software (Version 4.2.2; R Development Core Team, Vienna, Austria). The chi-square test was employed for categorical variables, and the Wilcoxon Rank Sum test was used for continuous variables in univariate analyses. Additionally, we implemented the Benjamini–Hochberg false discovery rate (FDR) correction to further control for false-positive results due to multiple testing. Generalized linear models are applied in multivariate regression analysis (specifically utilizing logistic regression for LVI). Spearman correlation was used to assess relationships between extratumoral sign subclassifications and prognostic factors. The R code used for all statistical analyses is available upon reasonable request from the corresponding author.

## 3. Results

### 3.1. Clinicopathological Features

The study included a total of 374 breast cancer patients presenting as NSNCM, consisting of 6 cases (1.6%) with grade I invasive ductal carcinoma, 227 cases (60.7%) with grade II invasive ductal carcinoma, and 141 cases (37.7%) with grade III invasive ductal carcinoma. The average age of the cohort was 53 ± 11 years (range: 27–79 years). Although the menopausal status was unknown, more than half of the patients (53.7%) were over the age of 50. Approximately 62.6% (*n* = 234) of the patients had tumors sized between 15 and 30 mm, and nodal metastasis was observed in 34.5% (*n* = 129) of them. Regarding hormonal receptors and HER2 subtypes, Luminal A accounted for a frequency of 57.8% (*n* = 216), followed by Luminal B (9.9%, *n* = 37), HER2-enriched (9.9%, *n* = 37), and TN subtype (22 0.4%, *n* = 84).

### 3.2. Identify the Focus Prognostic Factors

The Supporting Information Table S1 demonstrates statistically significant differences in mammographic appearances among LVI, Ki-67 levels, and sTIL levels (*p* < 0.01). Therefore, LVI, Ki-67 levels, and sTIL levels were identified as the focus prognostic factors.

### 3.3. Univariate Analysis for LVI, Ki-67 Levels, and sTIL Levels

The univariate analysis results of clinicopathological features, mammographic tumor signs, and extratumoral signs in malignant NSNCMs among LVI groups, Ki-67 groups, and sTIL groups are separately presented in Supporting Information Table S2.

In terms of clinicopathological features, only LN status and molecular subtypes exhibited significant variations between negative and positive LVI (*p*=0.001 and 0.037, respectively), while other features such as age, BMI, and CEA did not demonstrate statistically significant variations (*p* > 0.05). In the context of mammographic findings, there was no significant difference observed between the two groups of LVI in terms of tumor signs, including tumor shape, density, and margin (*p* > 0.05). However, a statistically significant difference was detected in extratumoral trabecular structural abnormalities between the groups (*p* < 0.001), while no notable variations were observed in the other two extratumoral signs (*p* > 0.05).

When analyzing the variations in clinicopathological features among different Ki-67 groups, we observed statistically significant differences across multiple features, including age (*p*=0.024), tumor size (*p*=0.008), histological grade (*p* < 0.001), LN status (*p*=0.041), and molecular subtypes (*p* < 0.001). Simultaneously, significant differences were noted in most mammographic extratumoral signs among malignant NSNCMs within these groups, such as extratumoral trabecular structural abnormalities (*p* < 0.001) and halo sign (*p*=0.002). However, no significant variations were noted in any tumor signs (*p* > 0.05).

The three groups of sTIL did not show any significant associations with all clinicopathological features (*p* > 0.05). Within the context of mammography observations, significant statistical differences were observed in extratumoral parenchymal structural abnormalities and tumor margin among the groups (*p* < 0.001 and *p*=0.013, respectively), while no significant variations were noted in the other tumor or extratumoral signs (*p* > 0.05).

### 3.4. Multivariate Logistic Regression Analysis for LVI

The logistic regression analysis results of variables associated with LVI are presented in Table 1. LN status emerged as the most robust independent predictor of LVI, exhibiting an odds ratio (OR) of 2.18 and a *p* value of 0.002. Additionally, extratumoral trabecular structural abnormalities demonstrated a significant association with LVI, displaying an OR of 2.5 and a *p* value of 0.007. Conversely, no independent association was observed between molecular subtypes and LVI (*p* > 0.05).

### 3.5. Correlation Analysis Between Subclassifications of Extratumoral Trabecular Signs and LVI

**Figure 3:**
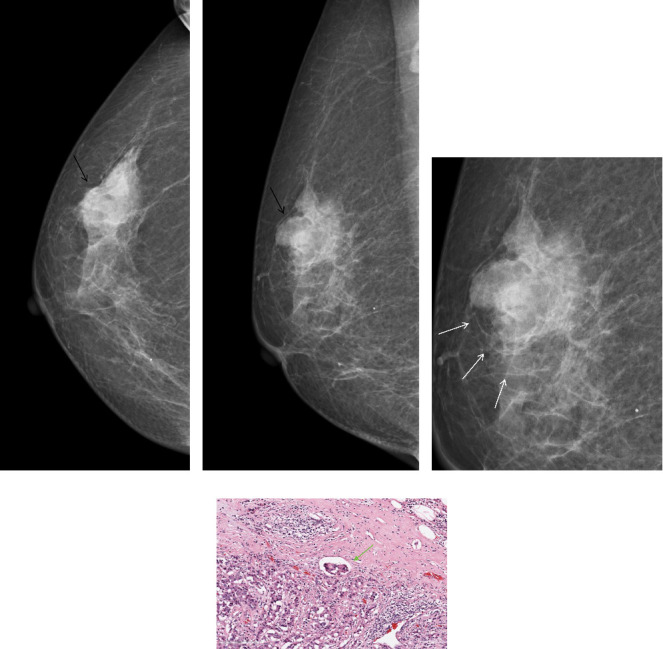
A 66-year-old woman proved to be invasive ductal carcinoma Grade III and LVI positive pathologically. The mammography images in the CC (a) and MLO (b) views revealed a mass located in the upper outer quadrant of the right breast (indicated by black arrow), with visible parallel trabecular structures surrounding it, (c) indicated by white arrow. Photomicrograph (H&E, × 200) of histologic specimen showed lymphovascular invasion, (d) indicated by green arrow.

The results presented in Table 2 demonstrate that among the three subclassifications of extratumoral trabecular structural abnormalities, namely parallel sign, vertical sign, and reticular sign, it is observed that the parallel sign exhibits a significant association with LVI and displays a positive correlation with LVI (*p* = 0.009, *r* = 0.134, Figure 3).

### 3.6. Multivariate Regression Analysis for Ki-67 Levels

The results of the multivariate regression analysis for variables associated with Ki-67 levels are presented in Table 3. Histological grade (OR = 2.10, *p* < 0.001) and extratumoral trabecular structural abnormalities (OR = 1.23, *p*=0.001) exhibited a significant correlation with the Ki-67 level.

### 3.7. Correlation Analysis Between Subclassifications of Extratumoral Trabecular Signs and Ki-67 Levels

**Figure 4:**
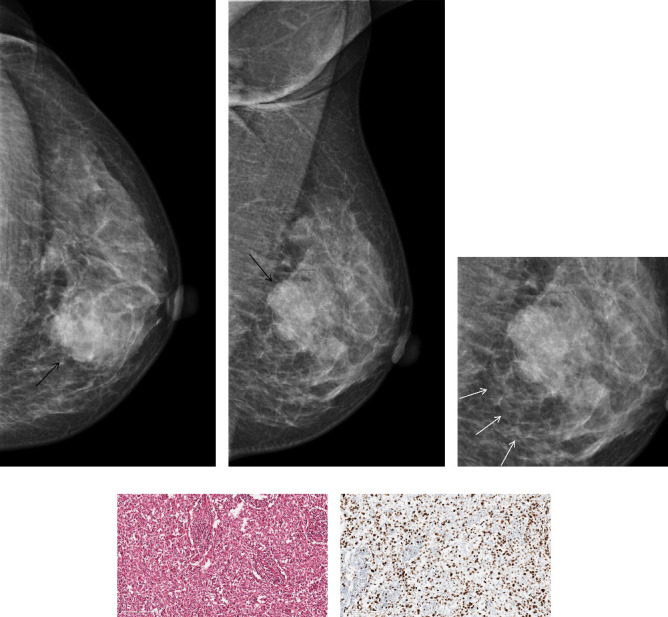
A 46-year-old woman proved to be invasive ductal carcinoma Grade III, high expression of Ki-67 pathologically. The mammography images in the CC (a) and MLO (b) views revealed the presence of a left breast mass (indicated by black arrow), accompanied by a surrounding reticular trabecular sign, (c) indicated by white arrow. Photomicrograph (H&E) (d) and IHC, (e) × 200 of histologic specimen showed that the Ki-67 proliferative index was >30%.

The positive correlation between the reticular sign and Ki-67 levels was observed among the three subclassifications of extratumoral trabecular structural abnormalities, as illustrated in Table 4 (*p*=0.009, *r* = 0.134, Figure 4).

### 3.8. Multivariate Regression Analysis for sTIL Levels

The multivariate regression analysis results for variables related to sTIL levels are displayed in Table 5. Extratumoral parenchymal structural abnormalities were identified as the strongest independent predictor of sTIL levels (OR = 0.64, *p* < 0.001), with tumor margin ranking second (OR = 0.74, *p*=0.028).

### 3.9. Correlation Analysis Between Subclassifications of Extratumoral Parenchymal Signs and sTIL Levels

**Figure 5:**
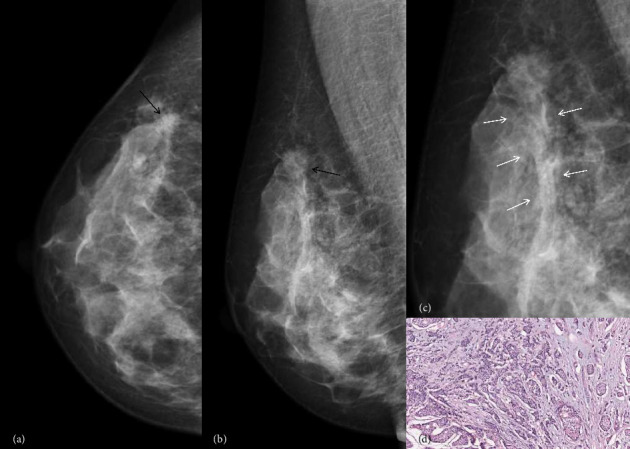
A 51-year-old woman proved to be invasive ductal carcinoma grade II, with low sTIL level pathologically. The mammography images in the CC (a) and MLO (b) views revealed a mass located in the upper outer quadrant of the right breast (indicated by black arrow), with visible extratumoral contraction sign of parenchyma ((c), indicated by white arrow). Photomicrograph (H&E, × 200) of histologic specimen showed sTILs < 10% (d).

**Figure 6:**
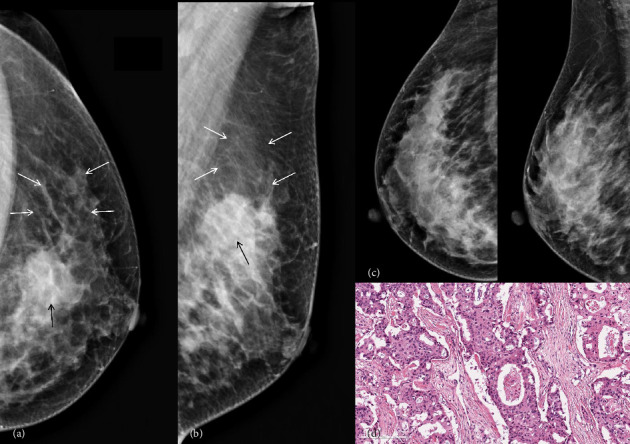
A 67-year-old woman proved to be invasive ductal carcinoma grade III, with low sTIL level pathologically. The mammography images in the CC (a) and MLO (b) views revealed the presence of a left breast mass (indicated by black arrow). With comparison to the contralateral breast (c), demonstrating parenchymal atrophy adjacent to the mass (indicated by white arrows). Photomicrograph (H&E, × 200) of histologic specimen showed sTILs < 10% (d).

The extratumoral parenchymal structural abnormalities can be classified into four subclassifications: contraction sign, distortion sign, pushing sign, and atrophy sign. Based on the findings presented in Table 6, both the contraction sign (*p* < 0.001, *r* = −0.185, Figure 5) and atrophy sign (*p*=0.046, *r* = −0.103, Figure 6) demonstrated a significant association with sTIL levels and exhibited a negative correlation with their levels.

## 4. Discussion

In our study, we aimed to identify detailed extratumoral signs and baseline tumor signs associated with prognostic factors observed in mammography of patients with malignant NSNCMs. The associations between traditional prognostic biomarkers and some extratumoral signs were found to be more significant than those between tumor signs and the same biomarkers. Consistently, the presence of extratumoral trabecular structural abnormalities emerged as an independent risk factor for LVI and Ki-67 expression, while the presence of extratumoral parenchymal structural abnormalities exhibited a significant correlation with sTIL levels. These findings are highly encouraging as they emphasize the necessity of simultaneously analyzing both tumor and extratumoral signs when assessing malignant NSNCM breast lesions using mammography.

Multiple studies have demonstrated that imaging characteristics can be influenced by factors such as tumor grade and hormone receptor status [7, 8]. Furthermore, various distinct mammographic appearances can effectively reflect the attributes and biological behaviors of tumors, thereby offering valuable insights to clinicians [26]. Consistent with previous investigations, our screening identified the focus prognostic factors that demonstrated an influence on mammographic features, including LVI, Ki-67 levels, and sTIL levels. Given that our study focused on NSNCMs and incorporated extratumoral signs, we observed no significant correlation with previously extensively investigated factors, such as the expression level of ER, PR, or HER2 [27, 28].

The peritumoral microenvironment, encompassing the extracellular matrix and diverse cellular components, plays a pivotal role in sustaining the wound response-like process while also contributing to inflammation, augmented vascular density, and enhanced permeability. This niche not only holds paramount significance for tumor progression but also harbors substantial prognostic potential [29, 30]. And the distinct microenvironment of breast carcinoma will inevitably show different signs in different imaging. In order to investigate the imaging-based alterations in the tumor microenvironment, we categorized the extratumoral signs of malignant NSNCMs into extratumoral structural abnormalities (parenchymal and trabecular) and halo sign. It is noteworthy that the BI-RADS lexicon [30] delineated architectural distortion in two dimensions. Firstly, it was defined as the absence of visible masses and the presence of thin straight lines radiating from a point, along with focal retraction, distortion, or lack of curvature at the edge of the parenchyma. Secondly, as an ancillary sign, it can indicate alterations in the structure surrounding the lesion. While prior investigations [31, 32] exclusively focused on architectural distortion while excluding masses, our study specifically scrutinized the structural deformation encompassing NSNCMs. Meanwhile, we also included conventional tumor signs in the study; however, the findings revealed that among the various tumor signs for malignant NSNCMs, only the tumor margin exhibited a significant correlation with the focus prognostic factors, while multiple extratumoral signs emerged as independent predictors of them. This observation further underscores the superior predictive value of extratumoral signs in assessing the prognosis of malignant NSNCMs.

The unfavorable outcomes observed in various malignancies have consistently been linked to the presence of LVI [33, 34]. In relation to breast cancer recurrence after modified radical mastectomy, the St. Gallen consensus on breast cancer has recognized lymphovascular tumor emboli, particularly lymphoneoplastic emboli, as a significant risk factor [35]. The study conducted by Liu et al. [36] demonstrated that the occurrence of LVI was not associated with demographic factors such as age, reproductive history, miscarriage history, family history of breast cancer, or other medical factors, which is consistent with our findings. Furthermore, their research provided support for the hypothesis that interstitial edema, subcutaneous fat blurring, and skin thickening are independent risk factors for LVI. Among them, the interstitial edema was defined as “the whole breast including subcutaneous fat layer is blurring, and multiple cord shadows are seen.” The definition of extramural trabecular structural abnormalities in the present study somewhat overlaps with this concept. Our findings suggested that extratumoral trabecular structural abnormalities, particularly the presence of parallel signs, exhibit a positive correlation with LVI. Hyperplastic fibrous tissue, dilated lymphatic vessels, or the ductal system can cause abnormal trabeculae. These parallel trabeculae were found surrounding the mass and can also be observed in deep fat or subcutaneous fat, even when not directly associated with the mass.

Additionally, several other studies have suggested a potential association between peritumoral edema and LVI [37]. Nevertheless, further research is required to ascertain whether this suggests that the extratumoral trabecular structural abnormalities represent a manifestation of peritumoral edema in malignant NSNCM digital mammographic images. Therefore, it is crucial for clinicians to remain vigilant when mammography reveals any abnormalities in the extratumoral trabecular structure or LNs, as these may indicate a higher likelihood of LVI.

The presence of extratumoral trabecular structural abnormalities was also identified as an autonomous risk factor for Ki-67 expression in this study. As a well-established marker of tumor proliferation, Ki-67 is associated with the tumor grade, recurrence, and response to neoadjuvant chemotherapy [38–41]. Our findings align with previous studies, such as those by Bocchi et al. [42] and Sun et al. [43], which demonstrated significant associations between the Ki-67 expression level and clinicopathological parameters including age, histological grade, tumor size, and mass visibility on mammography. Notably, Tamaki et al. [27, 28] observed that tumors with irregular shape, spiculated margins, and low density tended to have lower Ki-67 expression levels, which differs from our findings. This discrepancy may be attributed to differences in study design and inclusion criteria. Specifically, our study focused on NSNCMs and incorporated extratumoral imaging features, whereas Tamaki's study included a broader spectrum of tumor morphologies but did not assess the peritumoral microenvironment. The observed positive association between trabecular structural abnormalities and Ki-67 in our study may reflect underlying stromal remodeling and fibroblast proliferation associated with high-grade tumors.

However, all of these studies exclusively focused on the intrinsic and edge characteristics of tumor. To the best of our knowledge, there are few studies to compare extratumoral structural abnormalities in malignant NSNCMs with the expression level of Ki-67. The presence of reticular trabeculae exhibited a positive correlation with high Ki-67 expression in the present study. If we consider the potential correlation between parallel trabeculae and peritumoral edema, reticular trabeculae may serve as a more representative indicator of fibroblast proliferation. Due to fast mitosis, high-grade tumors develop more cell components and exhibit enhanced the peritumoral fibers proliferated. The overexpression of Ki-67 enhances cellular proliferation and increases malignancy. Therefore, extratumoral reticular trabecular structural abnormalities may be indicative of a higher malignant grade and higher expression of Ki-67.

Significant revelations have emerged regarding the pivotal role of TILs in governing the intricate dynamics of the tumor microenvironment [44–46], as they possess cytotoxic capabilities against tumor cells, particularly within specific subtypes of breast cancer [47]. The TILs can be categorized into intratumoral TILs (iTILs) and sTILs based on their spatial distribution either within the tumor cluster or within the tumor stroma [21, 23, 48]. Prior studies have demonstrated the superior reproducibility and prognostic value of sTILs compared to iTILs, especially in predicting response to neoadjuvant chemotherapy in breast cancer [23, 49]. Furthermore, Fang et al. [17] presented compelling evidence supporting the association between low sTIL levels and unfavorable disease-free survival (DFS), thereby highlighting its potential as a prognostic marker and offering a comprehensive perspective on the intricate prognostic landscape of breast cancer.

Previous studies have predominantly focused on specific subtypes of breast cancer when investigating the correlation between TIL levels and imaging features [50, 51]. For example, Ku et al. [50] conducted a study investigating the relationship between TIL levels and MRI features in TN breast cancer, proposing certain MRI characteristics of the tumor itself, such as round shape, circumscribed margin, homogeneous enhancement, and lack of multifocality, which were a major pattern of TN breast cancer with high TIL levels. The present study also yielded similar findings, suggesting that tumors with low and intermediate sTIL levels (96.7%) were more likely to exhibit indistinct margin compared to those with high sTIL (72.7%). Beyond tumor boundaries, we further observed that extratumoral parenchymal structural abnormalities, particularly the contraction sign, were the strongest imaging predictors of reduced sTIL levels. Specifically, we observed a negative association between the contraction sign and sTIL levels. Our previous study also confirmed a high positive predictive value (PPV) of parenchymal contraction sign in diagnosing malignant lesions [26]. The desmoplastic reaction has been considered as the host tissue's response to the tumor [52], while the contraction sign may primarily arise from extensive periductal fibrosis and elastic reaction [53]. This can be observed as a peritumoral or quadrantal parenchyma exhibiting a banded or “wedge-shaped” contraction, along with edge traction. The negative association between the contraction sign and sTIL levels may be mechanistically explained by the role of dense fibrotic stroma in creating an immune-excluded tumor microenvironment. Such fibrosis can serve as a physical barrier that limits lymphocyte infiltration and may also contribute to immune suppression through the activity of cancer-associated fibroblasts, which release immunomodulatory cytokines [54].

Similarly, parenchymal atrophy sign was also exhibited an inverse correlation with sTIL levels, which may reflect aggressive tumor expansion and depletion of adjacent stromal tissues, further hindering immune cell recruitment. To accurately evaluate atrophy signs, it is recommended to compare both breasts due to interindividual variations. In other words, the presence of parenchymal contraction sign or atrophy sign in malignant NSNCMs indicates lower levels of sTIL, which may indicate a poor prognosis.

In addition to the aforementioned features, our data accentuated traditional features, such as LN metastasis, and the histological grade remains crucial in the prediction of LVI and Ki-67 levels, separately.

Notably, though the associations were statistically significant, the effect sizes observed (e.g., OR = 0.64 for parenchymal abnormalities) indicate moderate predictive power. These imaging features may therefore serve as adjunctive markers for assessing tumor immune status, rather than definitive predictors on their own.

Our study had certain limitations. Firstly, it was a retrospective single-site study, which may not be representative of the general population. Moreover, the categorization of extratumoral structural abnormalities was based on visual assessment, which may introduce observer subjectivity. Although interpretations were performed by experienced radiologists, interobserver variability may still affect reproducibility. Future studies should include inter-rater agreement analysis and consider the application of standardized imaging criteria or AI-assisted diagnostic tools to reduce variation. Furthermore, this study lacked follow-up data. Obtaining long-term outcomes would provide additional evidence to substantiate the prognostic implications of extratumoral structural abnormalities. Multicenter studies are also warranted to refine and externally validate mammographic criteria for assessing extratumoral signs.

## 5. Conclusion

In conclusion, specific extratumoral structural abnormalities and their subclassifications in malignant NSNCMs showed a significant correlation with LVI, Ki-67, and sTIL levels. Given the prognostic value of these findings, we recommend that extratumoral signs be incorporated into structured mammographic reports to aid in risk stratification and preoperative decision-making. Careful evaluation of both tumor signs and extratumoral signs provides a more comprehensive imaging-based assessment and may serve as a valuable adjunct to limited histopathological sampling, particularly in challenging nonspiculated, noncalcified cases. Integrating these imaging features into clinical workflows has the potential to improve diagnostic accuracy and personalize breast cancer management strategies.

## Figures and Tables

**Table 1:** Multivariate logistic regression analysis for LVI.

Characteristics	OR	95% CI	*p* value
LN	2.18	1.33, 3.56	0.002^∗^
Molecular subtypes	1.13	0.85, 1.50	0.400
Trabecula	2.50	1.32, 5.09	0.007^∗^

Abbreviations: CI = confidence interval, LN = lymph node, and OR = odds ratio.

^∗^ *p* value < 0.05.

**Table 2:** Correlation analysis between subclassifications of extratumoral trabecular signs and LVI.

Characteristics	*p* value	*r*
Parallel sign	0.009^∗^	0.134
Vertical sign	0.191	0.068
Reticular sign	0.544	−0.031

*Note: r*: > 0 indicates a positive correlation; < 0 indicates a negative correlation.

^∗^ *p* value < 0.05.

**Table 3:** Multivariate regression analysis for Ki-67 levels.

Characteristic	OR	95% CI	*p* value
Age	1.00	0.99, 1.00	0.600
Tumor size	1.00	0.99, 1.00	0.300
Histological grade	2.10	1.88, 2.35	< 0.001^∗^
LN	1.05	0.94, 1.17	0.400
Molecular subtypes	0.97	0.91, 1.04	0.400
Trabecula	1.23	1.09, 1.39	0.001^∗^
Halo	1.05	0.94, 1.18	0.400

Abbreviations: CI = confidence interval, LN = lymph node, and OR = odds ratio.

^∗^ *p* value < 0.05.

**Table 4:** Correlation analysis between subclassifications of extratumoral trabecular signs and Ki-67 levels.

Characteristics	*p* value	*r*
Parallel sign	0.103	0.085
Vertical sign	0.921	0.005
Reticular sign	0.009^∗^	0.134

*Note: r*: > 0 indicates a positive correlation; < 0 indicates a negative correlation.

^∗^ *p* value < 0.05.

**Table 5:** Multivariate regression analysis for sTIL levels.

Characteristics	OR	95% CI	*p* value
Tumor margin	0.74	0.57, 0.97	0.028^∗^
Parenchyma	0.64	0.57, 0.71	< 0.001^∗^

Abbreviations: CI = confidence interval and OR = odds ratio.

^∗^ *p* value < 0.05.

**Table 6:** Correlation analysis between subclassifications of extratumoral parenchymal signs and sTIL levels.

Characteristics	*p* value	*r*
Contraction sign	< 0.001^∗^	−0.185
Distortion sign	0.053	−0.100
Pushing sign	0.305	−0.053
Atrophy sign	0.046^∗^	−0.103

*Note: r*: > 0 indicates a positive correlation; < 0 indicates a negative correlation.

^∗^ *p* value < 0.05.

## Data Availability

The data that support the findings of this study are available from the corresponding authors upon reasonable request.

## Nomenclature

NSNCM: Nonspiculate and noncalcified masses
LVI: Lymphovascular invasion
TILs: Tumor-infiltrating lymphocytes
MR: Magnetic resonance
PACS: Picture archiving and communication system
BMI: Body mass index
CEA: Carcinoembryonic antigen
CA153: Cancer-associated antigen 153
LN: Lymph node
ER: Estrogen receptor
PR: Progesterone receptor
PI: Proliferative index
IHC: Immunohistochemical
sTILs: Stromal tumor-infiltrating lymphocytes
iTILs: Intratumoral tumor-infiltrating lymphocytes
HER2: Epidermal growth factor receptor 2
LA: Luminal A
LB: Luminal B
TN: Triple negative
CC: Cranio-caudal
MLO: Medio-lateral oblique
ACR: American College of Radiology
BI-RADS: Breast imaging reporting and data system
OR: Odds ratio
CI: Confidence interval
DFS: Disease-free survival
PPV: Positive predictive value
H&E: Hematoxylin-eosin staining
NPI: Nottingham prognostic index
ASCO: American Society of Clinical Oncology
FISH: Fluorescence in situ hybridization

## Supporting Information

Supporting Information 1 Table S1: Univariate analysis between all prognostic factors and various mammography signs.

Supporting Information 2 Table S2: Univariate analysis across LVI, KI-67, and sTILs groups.
