# Supplementary material for: Extratumoral Signs of Malignant Nonspiculate and Noncalcified Masses on Mammography: Are They Associated With Prognostic Factors in Breast Cancer?
Source: Breast J. 2025 Jun 24;2025:2793342. doi: 10.1155/tbj/2793342 (PMC12213040; doi:10.1155/tbj/2793342)
Supplement: Supporting Information 1 — Table S1: Univariate analysis between all prognostic factors and various mammography signs. [file 2793342.f1.docx]

| **Clinicopathological Characteristics** |  | **Tumor shape** | | | | **Tumor density** | | | | **Tumor margin** | | | | **Parenchyma** | | | | **Trabecula** | | | | **Halo** | | |
| --- | --- | --- | --- | --- | --- | --- | --- | --- | --- | --- | --- | --- | --- | --- | --- | --- | --- | --- | --- | --- | --- | --- | --- | --- |
|  |  | **Round/Oval (n=308) N %** | **Irregular n=66 N %** | p -value | **Low/Equal (n=52) N %** | | **High (n=322) N %** | ***p -*value** | **Circumscribed/Obscured (n=15) N %** | | **Indistinct (n=359) N %** | ***p -*value** | **Negative  (n=111) N %** | | **Positive (n=263) N %** | ***p -*value** | **Negative  (n=89) N %** | | **Positive (n=285) N %** | ***p*- value** | **Negative (n=245) N %** | | **Positive (n=129) N %** | ***p -*value** |
| ER |  |  |  | 0.794 |  | |  | 0.496 |  | |  | 0.741 |  | |  | 0.316 |  | |  | 0.048 |  | |  | 0.124 |
|  | Negative | 106 (34.4%) | 21 (31.8%) |  | 15 (28.8%) | | 112 (34.8%) |  | 4 (26.7%) | | 123 (34.3%) |  | 33 (29.7%) | | 94 (35.7%) |  | 22 (24.7%) | | 105 (36.8%) |  | 76 (31.0%) | | 51 (39.5%) |  |
|  | Positive | 202 (65.6%) | 45 (68.2%) |  | 37 (71.2%) | | 210 (65.2%) |  | 11 (73.3%) | | 236 (65.7%) |  | 78 (70.3%) | | 169 (64.3%) |  | 67 (75.3%) | | 180 (63.2%) |  | 169 (69.0%) | | 78 (60.5%) |  |
| PR |  |  |  | 1.000 |  | |  | 0.640 |  | |  | 0.881 |  | |  | 0.773 |  | |  | 0.234 |  | |  | 0.280 |
|  | Negative | 119 (38.6%) | 25 (37.9%) |  | 18 (34.6%) | | 126 (39.1%) |  | 5 (33.3%) | | 139 (38.7%) |  | 41 (36.9%) | | 103 (39.2%) |  | 29 (32.6%) | | 115 (40.4%) |  | 89 (36.3%) | | 55 (42.6%) |  |
|  | Positive | 189 (61.4%) | 41 (62.1%) |  | 34 (65.4%) | | 196 (60.9%) |  | 10 (66.7%) | | 220 (61.3%) |  | 70 (63.1%) | | 160 (60.8%) |  | 60 (67.4%) | | 170 (59.6%) |  | 156 (63.7%) | | 74 (57.4%) |  |
| HER2^#^ |  |  |  | 0.593 |  | |  | 0.458 |  | |  | 0.100 |  | |  | 0.350 |  | |  | 0.012 |  | |  | 0.246 |
|  | Negative | 104 (33.8%) | 20 (30.3%) |  | 18 (34.6%) | | 106 (32.9%) |  | 7 (46.7%) | | 117 (32.6%) |  | 42 (37.8%) | | 82 (31.2%) |  | 18 (20.2%) | | 106 (37.2%) |  | 76 (31.0%) | | 48 (37.2%) |  |
|  | Low expression | 146 (47.4%) | 30 (45.5%) |  | 27 (51.9%) | | 149 (46.3%) |  | 8 (53.3%) | | 168 (46.8%) |  | 51 (45.9%) | | 125 (47.5%) |  | 51 (57.3%) | | 125 (43.9%) |  | 115 (47.0%) | | 61 (47.3%) |  |
| LVI |  |  |  | 0.934 |  | |  | 0.066 |  | |  | 0.539 |  | |  | 0.461 |  | |  | 0.008^*^ |  | |  | 1.000 |
|  | Negative | 233 (75.6%) | 49 (74.2%) |  | 45 (86.5%) | | 237 (73.6%) |  | 10 (66.7%) | | 272 (75.8%) |  | 87 (78.4%) | | 195 (74.1%) |  | 77 (86.5%) | | 205 (71.9%) |  | 185 (75.5%) | | 97 (75.2%) |  |
|  | Positive | 75 (24.4%) | 17 (25.8%) |  | 7 (13.5%) | | 85 (26.4%) |  | 5 (33.3%) | | 87 (24.2%) |  | 24 (21.6%) | | 68 (25.9%) |  | 12 (13.5%) | | 80 (28.1%) |  | 60 (24.5%) | | 32 (24.8%) |  |
| Ki-67 PI |  |  |  | 0.083 |  | |  | 0.210 |  | |  | 0.626 |  | |  | 0.673 |  | |  | <0.001^*^ |  | |  | 0.002^*^ |
|  | <10% | 24 (7.8%) | 9 (13.6%) |  | 8 (15.4%) | | 25 (7.7%) |  | 2 (13.3%) | | 31 (8.6%) |  | 12 (10.8%) | | 21 (8.0%) |  | 23 (25.8%) | | 10 (3.5%) |  | 19 (7.8%) | | 14 (10.9%) |  |
|  | 10%-30% | 145 (47.1%) | 36 (54.6%) |  | 23 (44.2%) | | 158 (49.1%) |  | 6 (40.0%) | | 175 (48.7%) |  | 52 (46.8%) | | 129 (49.0%) |  | 31 (34.8%) | | 150 (52.6%) |  | 135 (55.1%) | | 46 (35.7%) |  |
|  | >30% | 139 (45.1%) | 21 (31.8%) |  | 21 (40.4%) | | 139 (43.2%) |  | 7 (46.7%) | | 153 (42.7%) |  | 47 (42.4%) | | 113 (43.0%) |  | 35 (39.4%) | | 125 (43.9%) |  | 91 (37.1%) | | 69 (53.4%) |  |
| P53 |  |  |  | 0.160 |  | |  | 0.825 |  | |  | 0.725 |  | |  | 0.549 |  | |  | 0.790 |  | |  | 0.936 |
|  | Negative | 100 (32.5%) | 28 (42.4%) |  | 19 (36.5%) | | 109 (33.9%) |  | 4 (26.7%) | | 124 (34.5%) |  | 41 (36.9%) | | 87 (33.1%) |  | 32 (36.0%) | | 96 (33.7%) |  | 83 (33.9%) | | 45 (34.9%) |  |
|  | Positive | 208 (67.5%) | 38 (57.6%) |  | 33 (63.5%) | | 213 (66.1%) |  | 11 (73.3%) | | 235 (65.5%) |  | 70 (63.1%) | | 176 (66.9%) |  | 57 (64.0%) | | 189 (66.3%) |  | 162 (66.1%) | | 84 (65.1%) |  |
| sTILs |  |  |  | 0.395 |  | |  | 0.170 |  | |  | 0.013 |  | |  | <0.001* |  | |  | 0.379 |  | |  | 0.088 |
|  | 0-10% | 170 (55.2%) | 43 (65.2%) |  | 25 (48.1%) | | 188 (58.4%) |  | 7 (46.7%) | | 206 (57.4%) |  | 31 (27.9%) | | 182 (69.2%) |  | 53 (59.6%) | | 160 (56.1%) |  | 145 (59.2%) | | 68 (52.7%) |  |
|  | 11-39% | 128 (41.6%) | 22 (33.3%) |  | 24 (46.2%) | | 126 (39.1%) |  | 5 (33.3%) | | 145 (40.4%) |  | 74 (66.7%) | | 76 (28.9%) |  | 32 (36.0%) | | 118 (41.4%) |  | 96 (39.2%) | | 54 (41.9%) |  |
|  | ≥40% | 10 (3.2%) | 1 (1.5%) |  | 3 (5.7%) | | 8 (2.5%) |  | 3 (20.0%) | | 8 (2.2%) |  | 6 (5.4%) | | 5 (1.9%) |  | 4 (4.4%) | | 7 (2.5%) |  | 4 (1.6%) | | 7 (5.4%) |  |
| NPI grade^&^ |  |  |  | 0.284 |  | |  | 0.860 |  | |  | 0.294 |  | |  | 0.508 |  | |  | 0.099 |  | |  | 0.225 |
|  | 1 | 116 (37.7%) | 31 (47.0%) |  | 20 (38.5%) | | 127 (39.4%) |  | 9 (60.0%) | | 138 (38.4%) |  | 44 (39.6%) | | 103 (39.2%) |  | 44 (49.4%) | | 103 (36.1%) |  | 104 (42.4%) | | 43 (33.3%) |  |
|  | 2 | 181 (58.8%) | 32 (48.5%) |  | 31 (59.6%) | | 182 (56.5%) |  | 6 (40.0%) | | 207 (57.7%) |  | 65 (58.6%) | | 148 (56.3%) |  | 42 (47.2%) | | 171 (60.0%) |  | 132 (53.9%) | | 81 (62.8%) |  |
|  | 3 | 11 (3.5%) | 3 (4.5%) |  | 1 (1.9%) | | 13 (4.1%) |  | 0 (0.0%) | | 14 (3.9%) |  | 2 (1.8%) | | 12 (4.5%) |  | 3 (3.4%) | | 11 (3.9%) |  | 9 (3.7%) | | 5 (3.9%) |  |

ER: estrogen receptor; PR: progesterone receptor ; HER2: human epidermal growth factor receptor 2; LVI: lymphovascular invasion; PI: proliferative index; sTILs: stromal tumor-infiltrating lymphocytes; NPI: Nottingham prognostic index

^*^ *p*- value<0.01

^#^ HER2 levels were evaluated according to the latest American Society of Clinical Oncology(ASCO) guideline. HER2 negative: Immunohistochemical(IHC) 0; HER2 low expression: IHC 1+ or IHC 2+ with Fluorescence in situ hybridization (FISH)-negative; HER2 high expression: IHC 3+ or IHC 2+ with FISH-positive.

^&^ NPI was calculated according to the formula: NPI=size (cm)×0.2 + lymph node staging (1-3) + histologic classification (1-3). And NPI grade: 1(NPI<3.4), 2(NPI 3.4-5.4), 3(NPI>5.4).
