# Supplementary material for: Extratumoral Signs of Malignant Nonspiculate and Noncalcified Masses on Mammography: Are They Associated With Prognostic Factors in Breast Cancer?
Source: Breast J. 2025 Jun 24;2025:2793342. doi: 10.1155/tbj/2793342 (PMC12213040; doi:10.1155/tbj/2793342)
Supplement: Supporting Information 2 — Table S2: Univariate analysis across LVI, KI-67, and sTILs groups. [file 2793342.f2.docx]

| **Characteristics** |  | **Total** | **LVI** | | | **Ki-67 levels** | | | | **sTIL levels** | | | |
| --- | --- | --- | --- | --- | --- | --- | --- | --- | --- | --- | --- | --- | --- |
|  |  |  | **Negative** | **Positive** | ***p*-value** | **Low** | **Intermediate** | **High** | ***p*-value** | **Low** | **Intermediate** | **High** | ***p*-value** |
|  |  | **n=374** | **n=282** | **n=92** |  | **n=33** | **n=181** | **n=160** |  | **n=213** | **n=150** | **n=11** |  |
| Age (years) |  | 53 ± 11 | 52.0 [46.0;60.0] | 50.0 [46.0;59.2] | 0.515 | 48.0 [43.0;62.0] | 53.0 [47.0;61.0] | 50.5 [45.0;58.0] | 0.024^*^ | 52.0 [46.0;60.0] | 51.0 [45.0;59.0] | 54.0 [43.5;61.0] | 0.332 |
| BMI |  | 24.4 ± 4.0 | 24.2 [22.1;26.7] | 23.8 [22.0;27.4] | 0.697 | 24.0 [21.5;26.3] | 24.1 [22.4;26.8] | 24.1 [21.9;27.1] | 0.828 | 24.0 [22.0;26.7] | 24.0 [22.1;26.8] | 28.3 [23.9;29.0] | 0.147 |
| CEA (ng/ml) |  | 1.2 ± 0.1 | 1.9 [1.2;2.8] | 1.6 [1.3;2.9] | 0.784 | 1.6 [0.9;2.8] | 1.9 [1.2;2.9] | 1.8 [1.3;2.6] | 0.537 | 1.9 [1.3;2.9] | 1.6 [1.2;2.5] | 1.6 [1.2;2.0] | 0.064 |
| CA153 (U/ml) |  | 13.1 ± 9.8 | 10.8 [8.1;15.2] | 10.1 [7.4;15.8] | 0.472 | 9.7 [6.9;15.6] | 10.3 [7.6;15.2] | 10.9 [8.2;15.5] | 0.332 | 10.9 [7.72;16.0] | 10.2 [8.1;14.7] | 8.6 [7.0;16.2] | 0.729 |
| Tumor size (mm) |  | 22 ± 10 | 21.0 [16.0;27.0] | 20.0 [15.8;26.5] | 0.619 | 19.0 [14.0;28.0] | 19.0 [15.0;25.0] | 22.0 [17.0;30.0] | 0.008^*^ | 22.0 [16.0;29.0] | 19.5 [14.2;25.0] | 19.0 [18.0;22.0] | 0.068 |
| Histological grade |  |  |  |  | 0.588 |  |  |  | <0.001^*^ |  |  |  | 0.067 |
|  | G1-2 | 233 (62.3%) | 173 (61.3%) | 60 (65.2%) |  | 32 (97.0%) | 156 (86.2%) | 45 (28.1%) |  | 141 (66.2%) | 88 (58.7%) | 4 (36.4%) |  |
|  | G3 | 141 (37.7%) | 109 (38.7%) | 32 (34.8%) |  | 1 (3.0%) | 25 (13.8%) | 115 (71.9%) |  | 72 (33.8%) | 62 (41.3%) | 7 (63.6%) |  |
| LN |  |  |  |  | 0.001^*^ |  |  |  | 0.041^*^ |  |  |  | 0.060 |
|  | Negative | 245 (65.5%) | 198 (70.2%) | 47 (51.1%) |  | 26 (78.8%) | 108 (59.7%) | 111 (69.4%) |  | 131 (61.5%) | 104 (69.3%) | 10 (90.9%) |  |
|  | Positive | 129 (34.5%) | 84 (29.8%) | 45 (48.9%) |  | 7 (21.2%) | 73 (40.3%) | 49 (30.6%) |  | 82 (38.5%) | 46 (30.7%) | 1 (9.1%) |  |
| Molecular subtypes |  |  |  |  | 0.037^*^ |  |  |  | <0.001^*^ |  |  |  | 0.529 |
|  | LA | 216 (57.8%) | 158 (56.0%) | 58 (63.0%) |  | 29 (87.9%) | 130 (71.8%) | 57 (35.6%) |  | 129 (60.6%) | 83 (55.3%) | 4 (36.4%) |  |
|  | LB | 37 (9.9%) | 30 (10.6%) | 7 (7.6%) |  | 0 (0.0%) | 14 (7.7%) | 23 (14.4%) |  | 19 (8.9%) | 17 (11.3%) | 1 (9.1%) |  |
|  | HER2 | 37 (9.9%) | 23 (8.2%) | 14 (15.2%) |  | 3 (9.1%) | 19 (10.5%) | 15 (9.4%) |  | 21 (9.9%) | 15 (10.1%) | 1 (9.1%) |  |
|  | TN | 84 (22.4%) | 71 (25.2%) | 13 (14.2%) |  | 1 (3.0%) | 18 (10.0%) | 65 (40.6%) |  | 44 (20.6%) | 35 (23.3%) | 5 (45.4%) |  |
|  | LA | 216 (57.8%) | 158 (56.0%) | 58 (63.0%) |  | 29 (87.9%) | 130 (71.8%) | 57 (35.6%) |  | 129 (60.6%) | 83 (55.3%) | 4 (36.4%) |  |
| Tumor shape |  |  |  |  | 0.934 |  |  |  | 0.083 |  |  |  | 0.395 |
|  | Round/Oval | 308 (82.4%) | 233 (82.6%) | 75 (81.5%) |  | 24 (72.7%) | 145 (80.1%) | 139 (86.9%) |  | 170 (79.8%) | 128 (85.3%) | 10 (90.9%) |  |
|  | Irregular | 66 (17.6%) | 49 (17.4%) | 17 (18.5%) |  | 9 (27.3%) | 36 (19.9%) | 21 (13.1%) |  | 43 (20.2%) | 22 (14.7%) | 1 (9.1%) |  |
| Tumor density |  |  |  |  | 0.066 |  |  |  | 0.210 |  |  |  | 0.170 |
|  | Low/Equal | 52 (13.9%) | 45 (16.0%) | 7 (7.6%) |  | 8 (24.2%) | 23 (12.7%) | 21 (13.1%) |  | 25 (11.7%) | 24 (16.0%) | 3 (27.3%) |  |
|  | High | 322 (86.1%) | 237 (84.0%) | 85 (92.4%) |  | 25 (75.8%) | 158 (87.3%) | 139 (86.9%) |  | 188 (88.3%) | 126 (84.0%) | 8 (72.7%) |  |
| Tumor margin |  |  |  |  | 0.539 |  |  |  | 0.626 |  |  |  | 0.013^*^ |
|  | Circumscribed/Obscured | 15 (4.0%) | 10 (3.5%) | 5 (5.4%) |  | 2 (6.1%) | 6 (3.3%) | 7 (4.4%) |  | 7 (3.3%) | 5 (3.3%) | 3 (27.3%) |  |
|  | Indistinct | 359 (96.0%) | 272 (96.5%) | 87 (94.6%) |  | 31 (93.9%) | 175 (96.7%) | 153 (95.6%) |  | 206 (96.7%) | 145 (96.7%) | 8 (72.7%) |  |
| Parenchyma |  |  |  |  | 0.461 |  |  |  | 0.673 |  |  |  | <0.001^*^ |
|  | Negative | 111 (29.7%) | 87 (30.9%) | 24 (26.1%) |  | 12 (36.4%) | 52 (28.7%) | 47 (29.4%) |  | 31 (14.6%) | 74 (49.3%) | 6 (54.5%) |  |
|  | Positive | 263 (70.3%) | 195 (69.1%) | 68 (73.9%) |  | 21 (63.6%) | 129 (71.3%) | 113 (70.6%) |  | 182 (85.4%) | 76 (50.7%) | 5 (45.5%) |  |
| Trabecula |  |  |  |  | 0.008^*^ |  |  |  | <0.001^*^ |  |  |  | 0.379 |
|  | Negative | 89 (23.8%) | 77 (27.3%) | 12 (13.0%) |  | 23 (69.7%) | 31 (17.1%) | 35 (21.9%) |  | 53 (24.9%) | 32 (21.3%) | 4 (36.4%) |  |
|  | Positive | 285 (76.2%) | 205 (72.7%) | 80 (87.0%) |  | 10 (30.3%) | 150 (82.9%) | 125 (78.1%) |  | 160 (75.1%) | 118 (78.7%) | 7 (63.6%) |  |
| Halo |  |  |  |  | 1.000 |  |  |  | 0.002^*^ |  |  |  | 0.088 |
|  | Negative | 245 (65.5%) | 185 (65.6%) | 60 (65.2%) |  | 19 (57.6%) | 135 (74.6%) | 91 (56.9%) |  | 145 (68.1%) | 96 (64.0%) | 4 (36.4%) |  |
|  | Positive | 129 (34.5%) | 97 (34.4%) | 32 (34.8%) |  | 14 (42.4%) | 46 (25.4%) | 69 (43.1%) |  | 68 (31.9%) | 54 (36.0%) | 7 (63.6%) |  |

LA: Luminal A-like; LB: Luminal B-like; HER2: HER2-enriched; TN: Triple negative; LVI: lymphovascular invasion

^*^*p*-value < 0.05

Ki-67 levels: Low [Ki-67 proliferative index (PI) < 10%], Intermediate (10 < Ki-67 PI < 30%), High (Ki-67 PI > 30%)

sTIL levels: stromal tumor infiltrating lymphocyte levels, Low (sTILs ≤ 10%), Intermediate (10 < sTILs < 40%), High (sTILs ≥ 40%)
